# Child marriage, marital disruption, and marriage thereafter: evidence from a national survey

**DOI:** 10.1186/s12905-022-02088-z

**Published:** 2022-12-02

**Authors:** M. Mofizul Islam

**Affiliations:** Department of Public Health, La Trobe University, Room 410; Health Sciences Building 2, Melbourne, VIC 3086 Australia

**Keywords:** Marriage, Divorce, Remarriage, Child marriage, Age at first marriage, Multiple Indicator Cluster Survey

## Abstract

**Background:**

This study examines the relationship between women’s ages at their first marriages and the marital disruption among those who experienced child marriages and those who did not as well as identifies some compromises that women make in their remarriages after previous marital disruptions.

**Methods:**

The data of 57,476 women from the 2019 Bangladesh Multiple Indicator Cluster Survey were analysed using multivariable logistic and linear regressions. Women’s compromises in their remarriages were examined by determining the age differences with their current husbands, whether the current husband has another wife and their attitudes toward the justification of intimate partner violence by husbands.

**Results:**

Almost 65% of women experienced child marriage, and its prevalence is higher in rural (66.5%) than in urban areas (59.2%). The probability of marital disruptions decreases as the ages at the first marriages rise among women who experienced child marriages and increase among women who did not. Women living in rural areas are less likely than those living in urban areas to report marital dissolution (AOR 0.81, 95% CI 0.73–0.90). Also, women who completed relatively more years of education or have greater wealth are less likely to report marital disruptions and those who never gave birth are more likely to report these (AOR 3.54, 95% CI 3.14–3.99). Women who remarried after previous disruptions are more likely to report that their new husbands are, on average, almost 12 years older than they are, and have another wife. Also, those who experienced marital disruptions are more likely than others to believe that husbands are justified in beating their wives in certain circumstances.

**Conclusion:**

The odd of marital disruption decreases with the ages at first marriage among women who experienced child marriage and increase among women who did not. There is a curvilinear relationship between women’s ages at their first marriages and the probability of marital disruptions. Making compromises in remarriages after disruptions is common. Because marital disruption is increasing, appropriate policies are needed to address the adverse outcomes of divorces that ensue.

## Background

The age at which women enter their first marriages is a recognised determinant of the stability of their marriages [1]. Around four decades ago, researchers found evidence of a curvilinear (also known as U-shape) relationship between age at first marriage and marital dissolution [2]. The interpretation of this relationship is that both individuals who marry in their teenage years and those who marry after their late 20s would have higher risks of divorce. However, some studies conducted in recent years found a different pattern: marital dissolution declines as the women’s ages at their first marriages increase; in addition, this decline remains steady, and there is no upward turn in the curve [3–5]. These findings have brought into question the dominant understanding of the relationship between marital stability and age at first marriage. However, most of these recent studies were conducted in developed countries. Because of social and cultural differences, the relationship between age at first marriage and marital stability may be different in developing countries. In addition, in most developed countries, cohabitation before marriage is socially accepted, and this has a substantial effect on both the ages of women at their first marriages and marital stability [6–9]. In contrast, in many developing countries, particularly in patriarchal and religiously conservative societies, sexual intercourse outside marriage is considered to be a taboo, and cohabitation is not deemed acceptable. Also, individuals who are raised with no religion may be at a higher risk of marital disruption [10, 11] (note: the words disruption and dissolution have been used synonymously). Differences in race or ethnicity are other destabilizing factors [3, 12]. Given that the ages at first marriage and marital disruption are determined by a range of factors [13, 14], and many of which are socially embedded, it is unlikely that the results generated in developed countries will be applicable to developing countries.

Furthermore, few studies in the literature have examined the factors that affect marriage dissolution separately among women who experienced child marriages and those who did not. Women who enter child marriages often lack the opportunities to make informed choices and are being driven by their parents and/or the interests of men, at the exclusion of women’s agency [15]. Women in these marriages are also more likely to have lived in poverty, amidst gender inequalities and with harmful traditions, and all of these factors may have considerable effects on marital disruption [16]. In addition, there may be spatial variations in the prevalence of child marriages and the relationships between the women’s ages at first marriage and marital disruption that are due to variations in local customs and traditions. However, such spatial variations have rarely been accounted for. Moreover, the ways in which the women who experience divorce make compromises when they form new unions have not been explored.

### Theoretical framework

In literature, several theses try to explain the relationships between marital stability and the ages at first marriage [5]. The “earlier-is-better” is one such thesis that states that those who wait until they have completely developed their personal identities run risks of becoming rigid and not adapting to the needs and preferences of partners. Conversely, those who marry at younger ages do not have much time to solidify their individual identities and are more likely to develop couple identities, which facilitates marital stability [2]. Besides, these couples are unlikely to have extensive sexual histories that can act as comparison points to the current marital sexual relationships [5] and/or developing sexual attitudes that are incompatible with enduring marriages (e.g., non-monogamous sexual experiences) [17]. However, an antithesis to this theory is that marriages contracted during the teens may suffer from a “maturity effect”. Since people have insufficient knowledge at very young ages and are uncertain about their prospects and potential, they are prone to inappropriate partner selection [18]. In addition, some girls who marry relatively early may do so without parental blessing, creating a lack of parental and external social support when crises of marital stability arise [19].

The “later-is-better” or maturation thesis posits that individuals who marry younger have had little experience in looking for warning signs of future martial disruption and risk misjudging the characteristics of their prospective partners [20]. Also, delaying marriage means that the individuals leave hormones and strong sexual impulses behind and are therefore likely to find partners with similar sexual and long-term needs. Women who marry late tend to do so when they have completed schooling and can earn income [21], both of which are stabilizing factors. Also, according to the simple length of search thesis, the longer somebody searches for a partner and circulates in the marriage market, the higher the odds of finding a good match [5]. However, as time passes and women reach their late twenties or thirties while remain unmarried, they reduce their expectations and are willing to marry partners who are far from optimal. This may result in marital dissolution due to a poor match effect [20]. Moreover, at the later ages at marriage, there is greater exposure to the risk of having had a previous cohabitation and/ or a child from a previous partnership, which are destabilizing factors.

According to the “balanced-is-better” thesis, neither too young nor too old is good and delay is better only up to a point. According to this thesis, to enhance the chances of getting a good match, individuals should not be too young or too old, but rather should fall somewhere in between. Those who marry early may suffer from a maturity effect, and those who delay beyond the usual time have smaller pools for finding unmarried mates and settle for relatively poor matches. The “ticking of the biological clock” also pressures women, who wish to have children, to marry without further delay.

Women’s compromises in remarriage have been examined through the lenses of patriarchal bargaining [22]. In patriarchal conservative societies, after marital disruption or dissolution, usually women become dependent on parental families for material needs and social support. This dependence on others together with social stigma and blame for failing to sustain marriages put women in an extremely vulnerable situation [23]. To avoid this vulnerability, to acquire social status to match with social norms, and/or the provision of economic support with which to survive, some women enter into marriages again. Since their bargaining powers have been substantially reduced by having a history of marital dissolution, often they are ready to compromise in the selection of partners for remarriages. Also, after entering remarriages, women try hard to safeguard their remarriages even if they need to tolerate physical or emotional abuses from their husbands and in-laws [24, 25]. Thus, for many women, the second marriage is mostly a compromise.

As evident in the earlier sections, the relationship between age at marriage and the risk of marital dissolution is a complex phenomenon and it involves a diverse range of demographic, socio-cultural, economic and interpersonal dimensions. Also, women entering remarriages after a previous marital dissolution in a patriarchal society of low- and middle-income countries are likely to compromise in partner selection and safeguarding the new union. This study aimed to examine the association between the ages at first marriage and marital disruption among both those who experienced child marriages and those who did not and to identify some compromises that women make in their subsequent marriages.

## Materials and methods

### Study setting and data

Bangladesh, which is located in South Asia, is a developing country with a population exceeding 163 million. The population is predominantly Muslim. Marriage has always been a near-universal practice and is traditionally arranged by parents or relatives [26], although there is an increasing trend toward self-choice marriages among the current generation [27]. The legal age for marriage is currently 18 years for women and 21 years for men, although this law is widely ignored [26]. The average age at marriage has risen substantially over the past five decades. In Bangladesh, both husbands and wives have the right to initiate divorce. Although men initiate the vast majority of divorce cases, there has been an increasing tendency in recent years to have divorces that are initiated by women, especially in urban areas [28]. Maltreatment, infidelity, polygamy, impotency, infertility, substance use, women’s independence and modernisation and the financial incapacities of husbands are the most common reasons for divorce [13, 29, 30].

Data from the 2019 Bangladesh Multiple Indicator Cluster Survey (MICS) were used. The MICS are household surveys and implemented in countries under the programme developed by the United Nations Children's Fund (UNICEF) to provide nationally representative and rigorous data on children and women. The survey is validated and internationally recognised [31, 32]. MICS follows a two-stage stratified cluster sampling approach. The 2011 Bangladesh Census of Population and Housing was used as the sampling frame. At the first stage, the primary sampling units (PSUs) were selected from the census enumeration. The urban and rural areas within each of 64 administrative districts were identified as the main sampling strata. Within each stratum, a specified number of census enumeration areas were selected with probability proportional to size. Then a listing of households was carried out within the selected enumeration areas and 20 households from each PSU were drawn using random systematic selection procedures. Data were collected in face-to-face interviews with household members. The details of the sampling and data collection procedure of MICS can be found elsewhere [33]. Of the 64,400 households selected, 61,602 were found occupied and 61,242 were successfully interviewed (response rate 99%).

We defined child marriage as a formal marriage before women have reached 18 years of age. Women’s experiences of child marriages were identified from their ages at their first marriages. Their histories of marital disruption were identified by using the following two variables: their current marital statuses (currently married, formerly married or never married) and whether the women had been married only once or more than once. Those who had been widowed and had no history of marital dissolution at the time of the survey were not included in the group identified as “having a history of marital disruption”. Compromises in their remarriages and marital relationships among women who had married again after the dissolution of previous marriages were examined by using the following three variables: whether the age differences between them and their current husbands were very high, whether their current husbands had had another wife and whether the women believed their husbands were justified in violence against their intimate partners in certain circumstances. The attitudes toward justification of such violence were assessed using the following five circumstances: if the woman (1) goes out without telling her husband, (2) neglects the children, (3) argues with her husband, (4) refuses to have sex with her husband and (5) burns food while cooking.

### Analysis

The history of marriage disruption is the primary outcome, which is a binary variable. We used multivariable logistic regression to examine the relationship between ages at first marriage and marital dissolution. We also checked the interaction between the variables, ages at first marriage and child marriage. The regression models were adjusted for a range of potentially confounding variables that are available in the dataset. These variables include women’s ages at the time of the survey (in years), their educational statuses (none, primary, secondary, higher secondary or further), religions (Islam, Hindu or other), ethnicity (Bengali or other), whether they had ever given birth (yes or no), the wealth quintiles of their households (poorest, second, middle, fourth or richest), their locations (rural or urban) and place of residence (administrative divisions) (please see Table 1). The associations of regression were presented with adjusted odds ratios (AOR) and 95% confidence intervals (CIs). We used STATA version 15 for data analysis and Microsoft Excel for graphical presentation.

**Table 1:** Demographic and socio-economic characteristics of the participants

Variables	Married prior to 18 (n = 37, 279)	Married at 18 or later (n = 20, 197)	*p*
Had a history of marital disruption (%)	7.97	7.37	0.04
Age at first marriage	14.96 (SD ± 1.58)	20.34 (SD ± 2.86)	< 0.01
Women’s age	31.99 (SD ± 9.03)	27.36 (SD ± 9.73)	< 0.01
Women’s education (%)			
None	20.10	16.13	< 0.01
Primary	28.69	20.67	
Secondary	44.74	38.78	
Higher secondary or further	6.47	24.41	
Religion (%)			
Islam	90.58	84.49	< 0.01
Hindu	8.13	11.11	
Other	1.30	4.40	
Ethnicity (%)			
Bengali	98.66	95.56	< 0.01
Other	1.34	4.44	
Wealth quintile (%)			
Poorest	21.83	19.78	< 0.01
Second	22.75	17.78	
Middle	21.81	18.81	
Fourth	19.85	20.31	
Richest	13.76	23.32	
Ever given birth			
Yes	92.86	86.19	< 0.01
No	7.14	13.81	
Residence			
Urban	18.19	23.03	< 0.01
Rural	81.81	76.97	
Administrative region			
Barishal	9.00	8.59	< 0.01
Chattogram	15.54	22.57	
Dhaka	18.22	24.25	
Khulna	19.37	10.99	
Mymenshing	5.19	5.15	
Rajshahi	14.82	7.59	
Rangpur	13.06	10.68	
Sylhet	4.80	10.18	

The study also examined both the compromises made in the marriages and the marital relationships of those who had married again after previous marital dissolutions, using the t-test and chi-square test. The former test was used to examine the statistical significance in the age differences between the women and their current husbands and the latter to look at whether their current husbands have another wife and the women’s attitudes toward intimate partner violence by their husbands. We conducted linear regression to examine the relationships between the age difference between the women and their current husbands (dependent variable) and history of marital dissolution (independent variable). Logistic regression was conducted to estimate the odds ratio of whether the current husband has another wife, and women’s attitudes toward intimate partner violence. The attitude toward intimate partner violence was estimated using a composite score, which was developed based on women's responses to whether hitting or beating by one’s husband is justified in those five situations. Responses were coded as yes = 1 and no = − 1. Composite scores were computed for each respondent based on the average response score to the five items. So, the scores had a range from − 1 to 1. A high mean score indicates a weak rejection that is a strong justification for beating. Conversely, a low mean score indicates a strong rejection that is weak justification for beating. We then split the mean scores into two categories: (1) lenient or reject weakly (score 1–0) and (2) reject moderately to strongly (score 0 to − 1) These regression models were also adjusted for other covariates.

### Ethics statement

The MICS survey protocol was approved by technical committee of the Bangladesh Bureau of Statistics. Participants were informed of the voluntary nature of participation and the confidentiality and anonymity of information.

## Results

The dataset contains information on 57,476 women, of whom almost 65% had married before the age of 18, with the remaining 35% having married at 18 years of age or later (Table 1). The mean age at first marriage was 14.96 years among those who experienced child marriages compared to 20.34 years for those who were married at 18 years or later. At the time of the survey, the mean age of the women who had experienced child marriages was 32 years, which was significantly higher than the mean age of 27.36 years for those who had married at 18 or later. A larger proportion of those who had married at 18 or later had attained higher secondary or further education than of the women who had experienced child marriages. Child marriages were more prevalent among Muslim women than among those of other religions. Almost 82% of the women who had experienced child marriages were living in rural areas compared to 77% of those who had not. So, the prevalence of child marriage is higher in rural (66.5%; 30,498/46,044) than in urban areas (59.2%; 6,781/11,432) (These percentages are not shown in table). Table 1 shows the bi-variable relationships of child marriage to other variables. Almost 8% of the women who had experienced child marriages had histories of marital disruption compared to approximately 7.4% of those who had been married at the age of 18 or later.

Table 2 presents the results of multivariable regressions examining the association between marital dissolution and the ages at first marriage among women who had married before the age of 18 and those who had married at later ages. The interaction term of ages at first marriage and the variable *child marriage* is statistically significant. The odds of experiencing marital instability increase by a factor of 1.05 (95% CI 1.03–1.07) for per year increase in age at first marriage among women who did not experience child marriage, and by a factor of 0.91 (1.05 * 0.87) for every year increase if women experienced child marriage. The ages of the women at the time of the survey are also a significant positive predictor of marital dissolution (AOR 1.05, 95% CI 1.04–1.05). Education is a significant preventive factor for marital disruption, and there are trends showing decreased odds of marital disruption with increasing levels of education among both groups of women. In addition, women who belonged to the Hindu religion were significantly less likely to experience marital disruption than those who identified themselves as Muslims (AOR 0.54, 95%C: 0.46–0.63). Women from households of middle, upper-middle, or higher wealth statuses were significantly less likely than the poorest households to report marital. Furthermore, women who had never given birth were three and a half times likely to have experienced divorces. Also, women living in rural areas were less likely than those living in urban areas to report marital dissolution (AOR 0.81, 95% CI 0.73–0.90).

**Table 2:** Logistic regression examining the associations between age at first marriage and marital dissolution among women who did and did not experience child marriage

Variables	Marital dissolution (n = 53,931)	
	AOR (95% CI)	*p*
Child marriage		
No	1	
Yes	11.20 (6.32–19.85)	< 0.01
Age at first marriage	1.05 (1.03–1.07)	< 0.01
Age at first marriage # Child marriage^¶^	0.87 (0.84–0.90)	< 0.01
Women’s age at the time of the survey	1.05 (1.04–1.05)	< 0.01
Women’s education		
None	1	
Primary	0.76 (0.69–0.84)	< 0.01
Secondary	0.47 (0.42–0.52)	< 0.01
Higher secondary or further	0.38 (0.32–0.46)	< 0.01
Religion		
Islam	1	
Hindu	0.54 (0.46–0.63)	< 0.01
Other	0.64 (0.33–1.25)	0.19
Ethnicity		
Bengali	1	
Other	1.07 (0.48–2.38)	0.86
Wealth quintile		
Poorest	1	
Second	0.85 (0.77–0.95)	< 0.01
Middle	0.69 (0.61–0.77)	< 0.01
Fourth	0.72 (0.64–0.82)	< 0.01
Richest	0.63 (0.54–0.73)	< 0.01
Ever given birth		
Yes	1	
No	3.54 (3.14–3.99)	< 0.01
Residence		
Urban	1	
Rural	0.81 (0.73–0.90)	< 0.01
Administrative region		
Barishal	1	
Chattogram	1.53 (1.29–1.82)	< 0.01
Dhaka	1.37 (1.16–1.61)	< 0.01
Khulna	1.84 (1.56–2.16)	< 0.01
Mymenshing	1.27 (1.04–1.54)	< 0.02
Rajshahi	1.59 (1.35–1.88)	< 0.01
Rangpur	1.27 (1.07–1.51)	< 0.01
Sylhet	1.49 (1.23–1.80)	< 0.01
Constant	0.01 (0.006–0.02)	< 0.01

^¶^Interaction term

**Fig. 1:**
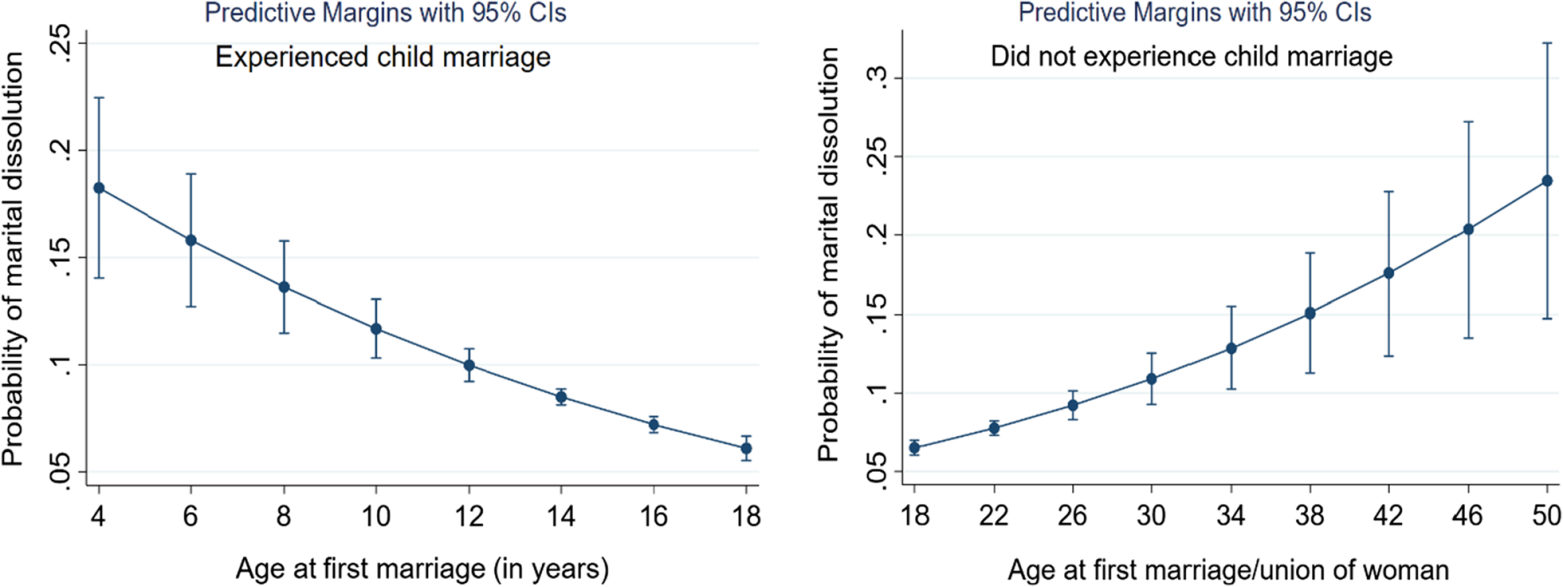
Marginal plot showing the relationship between age at first marriage and the probability of marital dissolution among women who did and did not experience child marriage

Figure 1 presents the relationship between the ages at first marriage and the probability of marital dissolution. The probability of a history of marital dissolution decreases gradually as the ages at first marriage rise among women who experienced child marriages. The probability of dissolution increases as the ages at their first marriages increase among women who did not experience child marriages. This relationship is consistent with the results presented in Table 1. These two plots, together, indicate a possible curvilinear (U-shape) relationship between age at first marriage and the probability of marital dissolution.

Table 3 illustrates the compromises that women make in later marriages after having experienced divorce. These women were more likely to have married men who were considerably older than they were (mean age difference = 11.84 years) and to report that their husbands had had another wife. Women who had histories of divorce were significantly more likely than women who had had stable relationships to believe that husbands were justified in beating their wives in certain circumstances, as shown in Table 3. Among women with histories of divorce, those who were married at the time of the survey were more likely to believe that their husbands were justified in this than those who were not married. Among women with histories of previous marital disruptions, almost 39% reported being in married relationships at the time of the survey, meaning that they had remarried (results not shown in the table).

**Table 3:** Bi-variate associations between the history of marital dissolution and some compromises women do in their marriages thereafter

Variables	Stable (A)	Experienced dissolution (B)	Experienced dissolution		*p* (A vs B)	*p* (C vs D)
			Currentlymarried (C)	Currently unmarried (D)		
Mean age difference with the current husband (years)	7.80	11.84	11.84	–	< 0.01	–
Current husband has another wife (%)	2.41	27.09	27.09	–	< 0.01	–
*Attitudes towards intimate violence by husbands* Intimate violence by husband is … …						
Justified if she goes out without telling her husband (%)	14.79	17.04	20.24	14.99	< 0.01	< 0.01
Justified if she neglects the children (%)	16.30	18.19	21.79	15.89	< 0.01	< 0.01
Justified if she argues with her husband (%)	22.57	24.80	30.52	21.17	< 0.01	< 0.01
Justified if she refuses to do sex with her husband (%)	10.17	12.48	14.42	11.22	< 0.01	< 0.01
Justified if she burns food while cooking (%)	6.58	8.78	9.07	8.56	< 0.01	0.58

The multivariable regression concurs with the bivariate results on compromises women make in their remarriages (Table 4). In their remarriages, these women were more likely to have married men who were considerably older than they were, compared to women who did not experience marital dissolution. The mean age of current husbands was almost four years higher (95% CI 3.44–4.46) for women now married but had a history of marital dissolution than the mean age of husbands of married women who did not experience marital dissolution. Similarly, women who experienced marital dissolution were almost 12 times likely than women who did not experience it to report that their current husbands had another wife. Likewise, currently married women with a history of marital dissolution showed significantly more leniency towards intimate partner violence (AOR 1.18, 95% CI 1.02–1.37).

**Table 4:** Multivariable regression between the history of marital dissolution and some compromises women do in their marriages thereafter

	Mean age difference with the current husband ACoef (95% CI)	Current husband has another wife AOR (95% CI)	Attitudes towards intimate partner violence AOR (95% CI)
Experienced dissolution and currently married	3.95 (3.44–4.46)	11.81 (10.08–13.83)	1.18 (1.02–1.37)

Adjusted coefficient (ACoef) and adjusted odds ratios (AORs) of covariates are not presented

## Discussion

The findings of this study suggest that in Bangladesh, the odds of marital dissolution decrease with age at first marriage among women who experienced child marriage. On the other hand, the odds of marital dissolution increase with age at first marriage among women who did not experience child marriage. Together, it appears that the relationship between marital disruption and women’s ages at their first marriages is curvilinear. Thus, our results support the “balanced-is-better” approach, as it may offer couples better marital stability. The marriage disruption rate was higher among women who had experienced child marriages than those who had not. The likelihood of marital disruption decreased with increasing levels of education and household wealth. Also, women who had ever given birth were significantly less likely to experience marital disruption. The mean age difference between women and their current husbands was significantly higher in women’s remarriages than in their first marriages. Findings also suggest women who remarried were more likely to report that their husbands had another wife. Women who had histories of disruption were significantly more likely than women who had stable relationships to believe that husbands are justified in beating their wives in certain circumstances. Among women with histories of disruption, those who were in marital relationships (i.e., remarried) at the time of the survey were more likely to approve the justification than those who were not.

Bangladesh is known for the near universality of marriage and the early ages of entry into marital unions. Although the legal marriage age for women is 18 years, we found that almost 65% of marriages occurred before this age, and this rate is consistent with the census data [26]. The practice of child marriages, particularly among girls, is socially and culturally embedded in society. Religious and societal restrictions regarding sexual relationships before marriage, along with poverty, lack of access to education and the insecurities unmarried girls face, are also vital factors that influence child marriages [34]. Our findings suggest that marital disruptions significantly decrease as the ages at first marriages increase among women who experienced child marriages. Some possible reasons for this have been mentioned in the earlier sections. In addition, girls of very young ages may not be physically and psychologically mature enough to cope with the biological demands of their husbands, to have successful pregnancies and to be able to handle the heavy load of household work [35]. With the growing awareness of the adverse outcomes associated with child marriages [36] and the introduction of strict laws, child marriages have been gradually decreasing in Bangladesh; indeed, the prevalence dropped from 90% around 1970 to just over 50% in 2020 [37]. However, the current prevalence remains very high in comparison to many other developing countries. Child marriages also obstruct education, since around 75% of girls drop out of school after their marriages [38].

Although divorce may be a better outcome for women in some circumstances, marital stability is a key indicator of well-being and is linked to the mental and physical health of the couples [39]. After experiencing marital disruptions, most women in Bangladesh return to their parental homes and become dependent on their families. In resource-poor families, divorced women are usually considered to be burdens, mainly because many do not have formal jobs. Although women have some entitlements after marital dissolution, including contract money and maintenance to support themselves and their children, these are hardly ever realised. When children remain with their fathers, they often suffer from neglect and maltreatment by stepmothers if their fathers have remarried. In addition, communities look down on women who have experienced marital dissolutions and on their children [35]. Consequently, marital disruption often brings negative repercussions for children's upbringings, health and well-being and academic and social performances [40–42]. Thus, at the population level, marital disruption is a public health issue [43]. While removing all factors that cause marital dissolution is unattainable in the short run, some factors such as reducing child marriage and ensuring education for girls are achievable targets. Recent initiatives in Bangladesh regarding legal and social aspects of child marriage and universal education for girls are likely to bring noticeable changes.

However, the significantly increasing rate of divorce in Bangladesh in recent years is of great concern [29]. The literature suggests that several socio-economic factors have caused this, including improvements in women's social statuses, their rising participation in the formal workforce, individualism, modernization, higher tolerance for divorce [13, 29, 30]. Indeed, divorce behaviour appears to be changing in Bangladesh, with more women initiating divorce petitions [29]. There has been a steep rise in divorce cases, particularly in urban areas, which is consistent with our result. During the COVID-19 lockdown period, divorce has substantially increased in city areas [28]. Divorced women face difficulties with basic subsistence, social stigma, exclusion, harassment in the workplace and psychological disturbances [44]. Moreover, they risk sexual assault, particularly if they are relatively young. In addition, the dominant social and religious belief in Bangladesh is that the marital relationship is sacred, and that women should not stay single. All these socio-economic factors create pressure on divorced women to remarry [45]. While remarriage after divorce is common in developed countries, in a patriarchal society like Bangladesh, it is a difficult task. Since the supply of potential partners is low, divorced women must often compromise, settling for less desirable partners. As our results suggest, these women may also take relatively lenient approaches to intimate partner violence, which can probably be attributed to the added pressure to succeed after a previous marital failure.

This study has some limitations. Many factors other than the ages at first marriage influence the likelihood of marital dissolution [14, 46, 47], but we do not have the necessary data for adjusting our models for all those variables. One such variable is the duration of a marital relationship, which is a potential risk factor for marital stability [20]. In addition, we do not know whether our participants’ families had histories of marital dissolution, so there may have been intergenerational transmission of divorce [48, 49]. Furthermore, in Bangladesh, desirable traits in brides are their physical beauty, and for grooms it is their wealth and earning capacities [13]. These traits may be associated with marital stability; however, we have no data on this. In women's remarriages, the current husbands were more likely to have another wife, but we do not know if the husband already had a wife or married another woman later. Finally, as this is a cross-sectional study, we cannot investigate temporal relationships between independent and outcome variables and eliminate the possibility of reverse causality.

In the context of Bangladesh, the “balanced-is-better” approach appears to work better than the “earlier-is-better” or “later-is-better” approaches with regard to the relationship between age at first marriage and marital stability. Child marriages are common in Bangladesh. A higher proportion of women who experienced child marriages reported marriage disruptions than those who did not. Women who remarry after previous marital disruptions appear to compromise in selecting husbands. As the rate of divorce is rising, appropriate social policies are needed to reduce the adverse outcomes that divorced women face.

## Data Availability

The data that support the findings of this study are publicly available on the website of UNICEF MICS upon its approval.
